# Differential expression profiles of microRNAs in musk gland of unmated and mated forest musk deer (*Moschus berezovskii*)

**DOI:** 10.7717/peerj.12710

**Published:** 2021-12-22

**Authors:** Hang Jie, Zhongxian Xu, Jian Gao, Feng Li, Yinglian Chen, Dejun Zeng, Guijun Zhao, Diyan Li

**Affiliations:** 1 Chongqing Institute of Medicinal Plant Cultivation, Bio-resource Research and Utilization joint key laboratory of Sichuan and Chongqing, Nanchuan, Chongqing, China; 2 Sichuan Agricultural University, Institute of Animal Genetics and Breeding, College of Animal Science and Technology, Chengdu, Sichuan, China; 3 China West Normal University, Key Laboratory of Southwest China Wildlife Resources Conservation (Ministry of Education), Nanchong, Sichuan, China

**Keywords:** MicroRNA, Musk deer, Musk gland, Expression profile, Mating status

## Abstract

**Background:**

The formation of musk is a complex biophysical and biochemical process that change with the rut of male forest musk deer. We have reported that the mating status of male forest musk deer might result to the variations of chemical composition and microbiota of musk and its yields. Critical roles for microRNAs (miRNAs) of multi-tissues were profiled in our previous study; however, the role for miRNAs of the musk gland remains unclear in this species.

**Methods:**

In this study, we used Illumina deep sequencing technology to sequence the small RNA transcriptome of unmated male (UM) and mated male (UM) of Chinese forest musk deer.

**Results:**

We identified 1,652 known miRNAs and 45 novel miRNAs, of which there were 174 differentially expressed miRNAs between UM and MM. chi-miR-21-5p, ipu-miR-99b and bta-miR-26a were up-regulated in UM among the 10 most differentially expressed miRNAs. Functional enrichment of the target genes showed that monosaccharide biosynthetic process, protein targeting, cellular protein catabolic process enriched higher in MM. Meanwhile, structural molecule activity, secretion by cell, regulated exocytosis and circulatory system process enriched more in UM, hinting that the formation of musk in UM was mediated by target genes related to exocytosis. The miRNA-mRNA pairs such as miR-21: *CHD7*, miR143: *HSD17B7*, miR-141/200a: *Noc2* might involve in musk gland development and musk secretion, which need to be verified in future study.

## Introduction

Chinese forest musk deer is an economically important species that is mainly distributed in western China. The musk produced by the male musk gland has a unique perfume and pharmaceutical characteristics, so is often used as a raw material for perfumery and Asian traditional medicine (Meng et al., 2012; Shrestha, 1998). However, the musk deer population has drastically declined in recent decades through overhunting, causing it to be listed as an endangered species on the IUCN Red List (Yang et al., 2003). Musk deer are seasonal breeders, and the rut of the male musk deer starts in October and extends to February of the following year. Furthermore, the formation of musk is also seasonal, the musk-secreting period is usually during May to July and the maturation of musk is usually in August and September (Zhang et al., 2015). The formation of musk is a complex biophysical and biochemical process involving microorganisms. Age, nutrition, and the level of sex hormones of the male musk deer have been reported to affect the musk yield (Zhang, 1983; Chen, Ma & Zhou, 1992; Bai et al., 2013). Additionally, we previously showed that unmated male musk deer (22.79 ± 3.6 g, *n* = 5) secrete more musk than mated males (1.12 ± 0.34 g, *n* = 5) (Li et al., 2016). We found 137 differentially expressed genes between unmated and mated sexually mature Chinese forest musk deer in our previous study. The “PPAR signaling pathway”, “Calcium signaling pathway”, “PI3K-Akt signaling pathway” and “Thyroid hormone synthesis” pathways were more abundant in unmated musk deer (Jie et al., 2019). In addition, we found the serum testosterone levels in unmated males were much higher than that of mated males, especially during the vigorous musk secretion period (69.44 ± 3.24 nmol/L in unmated males and 57.24 ± 6.65 nmol/L in mated males, *P*<0.05, *n* = 10) (our unpublished data). However, the molecular mechanism underlying the relationship between mating status and musk yield is unclear.

MicroRNAs (miRNAs) are noncoding, endogenous small RNAs (approximately 22 nucleotides in length) that regulate gene expression by binding to the 3′ untranslated region (3′ UTR) of mRNAs (Ambros, 2004). They are evolutionarily conserved in bilaterian animals although the fact that the current nomenclature conventions make annotation rather subjective in accepting putative new miRNA-related sequences (Wilczynska & Bushell, 2015; Takane et al., 2010; Desvignes et al., 2015). The miRNA-mediated repression of gene expression occurs either through the degradation of target mRNAs or the repression of target mRNA translation (Yekta, Shih & Bartel, 2004; Nilsen, 2007). In metazoans, most miRNAs negatively regulate protein expression by binding to the 3′ UTRs of target mRNAs with imperfect complementarity, but have no influence on mRNA levels (Catalanotto, Cogoni & Zardo, 2016). Increasing evidence has demonstrated that miRNAs exhibit spatiotemporal expression patterns, and that they participate in the regulation of a range of biological processes such as cell proliferation and differentiation, organ development, and tumorigenesis (Ambros, 2004; Hwang & Mendell, 2006; Krichevsky et al., 2003). Recently, they were also shown to be involved in the development of gonads and the secretion of sex hormones in mammals. Bjork et al. (2010) reported that miR-18 mediates male germ cell maturation by directly targeting heat shock factor 2 in mouse, while miR-17-92 and miR-106b-25 were shown to regulate spermatogonial differentiation in mice (Tong et al., 2012). However, the role of miRNAs in the musk gland of male musk deer, as an accessory sex gland, has not been explored.

In this study, we sequenced the small RNA transcriptomes of the musk gland of unmated and mated musk deer using Illumina high-throughput sequencing. Our results will help to better comprehend the roles of miRNAs in musk gland of forest musk deer.

## Materials & Methods

### Collection of musk gland

Forest musk deer raised in Chongqing Institute of Medicinal Plant Cultivation (Chongqing, China, altitude: 678 m). Every individual was housed in a 12 m^2^ single living room, there were a big play ground in front of 5 living rooms. The concentrate-to-forage ratio of feed was 1:4. The coarse fodders were the plants harvested from the area nearby, including *Ipomoea batatas*, *Pittos porum* tiny leaves, *Broussonetia papyrifera* tiny leaves, and other light green weeds. The concentrates was concluded 65% corn, 25% full-fat soybean, 6% wheat bran, 0.4% NaCl, 1.5% CaCO_3_, 0.8% CaHPO_4_, 0.15% muti-vitamin, 0.15% muti-mineral. *Pittos porum* and *Broussonetia papyrifera* tiny leaves were fed at 08:30 every morning and a mixture of concentrates and other coarse fodders were fed at 17:30 every afternoon. And forest musk deer could drink water freely. HIKVISION video cameras were applied to monitored the mating behavior (Li et al., 2016). According to the mating situation records, musk gland tissues were collected from unmated (*n* = 1) and mated (*n* = 1) musk deer at the age of ∼4.5 which died from earthquake at April 20th, 2013. (Animals that suffered the earthquake did not die immediately, they died from incurable injuries, during which we prepared all stuffs.) Autopsy was conduct within 5 min after its death, the musk gland was dissected and quickly stored in sterile cryopreservation tubes with RNAlater (Ambion), and immediately frozen with liquid nitrogen before transferred it to −80 °C until total RNA extraction. Experimental procedures were approved by the Institutional Animal Care and Use Committee of Sichuan Agricultural University (permit number: S20151006) and all efforts were made to minimize suffering.

### Small RNA library construction and high-throughput sequencing

Total RNA from each sample was extracted with TRIzol (Invitrogen, Carlsbad, CA) following the manufacturer’s instructions. The quantity and integrity of RNA were examined using an Agilent 2100 bioanalyzer (Agilent Technologies, Palo Alto, CA) and 1% agarose gel electrophoresis. Total RNA (∼20 µg) from the musk glands of the UM and MM was used to construct library, respectively. Small RNAs of 17–35 nt fractions was enriched from these corresponding libraries of UM and MM using polyacrylamide gel electrophoresis, then ligated to adaptors. Subsequently, the adaptor-ligated small RNAs were reverse transcribed into cDNA and amplified using 15 PCR cycles. Finally, TruSeq^®^ Small RNA Library Prep Kit (Illumina) was used to construct the small RNA libraries, subsequently sequenced by a 50-bp single end reads (SE-50) on the Illumina HiSeq 2000 platform at Sangon Biotech (Shanghai, China). Raw data of Illumina sequencing have been deposited in NCBI with to the SRA (Short Read Archive) accession number: No. SRA548312 (https://www.ncbi.nlm.nih.gov/sra/?term=SRA548312) which contain 2 libraries SRR5378549 and SRR5366118.

### Basic analysis of small RNA sequencing data

After high-throughput sequencing, the 3′ adaptor sequences were removed using Cutadapt software (version 1.9.1) (Martin, 2011), and truncated reads longer than 35 nt or shorter than 17 nt in length were removed. FASTX-Toolkit software (version 0.0.14) (http://hannonlab.cshl.edu/fastx_toolkit/) was used to filter the remaining reads, discarding those with ≥20% of bases with a Phred score <30 using a Fastq quality filter (−q 30 −p 80). Fastx collapse was then used to remove identical reads. To annotate the small RNAs(tRNA, rRNA, small nuclear (sn)RNA, small nucleolar (sno)RNA, small conditional (sc)RNA, and repeats), remaining reads were compared against tRNAdb (http://trna.bioinf.uni-leipzig.de/) (Jcohling et al., 2009), SILVA rRNA (https://www.arb-silva.de/) (Quast et al., 2013), GenBank (https://www.ncbi.nlm.nih.gov/genbank/) (Benson et al., 2013) and RepBase (http://www.girinst.org/repbase/) (Bao, Kojima & Kohany, 2015) databases using Bowtie v.1.0.0 software (Langmead, 2010). Because no information exists about Chinese forest musk deer miRNAs, the unannotated reads were compared against known metazoan mature miRNAs obtained from miRBase 21.0 (http://www.mirbase.org/) (Kozomara & Griffithsjones, 2014) to identify known miRNAs. Only reads perfectly matched with miRBase were considered as known miRNAs. The unmatched reads were aligned with the integrated transcriptome of musk deer to predict novel miRNAs by miRDeep2 software (Friedländer et al., 2008). Only reads whose hairpin structure had a significant randfold *P*-value (<0.05) and miRDeep score higher than 5 could be considered as novel miRNAs.

### Differential expression analysis of known miRNAs

To identify known miRNAs that were differentially expressed between unmated and mated musk deer, miRNA reads were normalized using tags per million ((TPM) = Actual miRNA count/Total clean reads count*1,000,000). When the TPM value of a particular miRNA is ≤1 in two groups, this miRNA cannot be used to perform further differential expression analysis. Then, the DEGseq R package (http://www.bioconductor.org/packages/release/bioc/html/DEGseq.html) (Wang et al., 2010) was used to calculate fold-change and *P*-value based on normalized expression values. Only miRNAs with fold-changes ≥2 and *P*-values <0.001 were defined as differentially expressed.

### Prediction of target genes of differentially expressed miRNAs

MiRanda3.3a (Enright et al., 2003) and RNAhybrid2.2.2 (Krüger & Rehmsmeier, 2006) software were used to identify putative target genes of differentially expressed miRNAs by aligning them with the reference genes of musk deer annotated in our previous study (Jie et al., 2019). In miRanda, putative target genes were filtered with a threshold of >150 and a minimum free energy of <−20 kcal/mol. Human 3 ′ UTRs were consulted to predict target genes, and RNAhybrid parameters were set as follows: (1) minimum free energy <−25 kcal/mol; and (2) maximum internal loop size of four nucleotides. Genes commonly predicted by both types of software were defined as target genes.

### Functional enrichment of putative target genes of the top 10 expressed miRNAs in UM and MM

We selected putative target genes of the 10 most highly expressed miRNAs of UM and MM respectively to perform functional enrichment using metascape with default parameters (Zhou et al., 2019). The details were as follows: Initially, we chose “Customer Analysis” to avoid redundancy of multiple annotated databases. We selected “GO Molecular Functions” and “GO Biological Processes” for functional enrichment, with the default parameters of minimum enrichment: 1.5, *P* valve cutoff: 0.01.

## Results

### Overview of sequencing data

**Figure 1:**
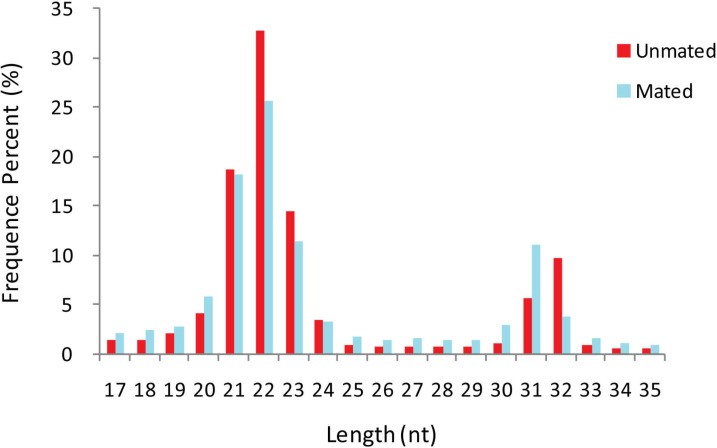
Length distribution-revised.

A total of 26,179,144 and 23,573,546 raw sequences were obtained from unmated (*n* = 1) and mated (*n* = 1) groups, respectively. After removing adaptor sequences, truncating reads longer than 35 nt or less than 17 nt, and low-quality sequences, a total of 19,435,154 and 16,818,354 clean reads (including 1,206,318 and 1,518,258 unique reads) were obtained, respectively (Table S1). The length distribution of the total clean reads in the two libraries was similar (Fig. 1). We found that the majority of small RNAs were 20–24 nt long, and that the most abundant reads were 22 nt, which accounted for 32.82% and 25.65% in unmated and mated libraries respectively, followed by 21 nt (18.61% and 18.21%), and 23 nt (14.52% and 11.40%). Another small peak distribution at 31–32 nt also accounted for a higher proportion in the two libraries.

**Figure 2:**
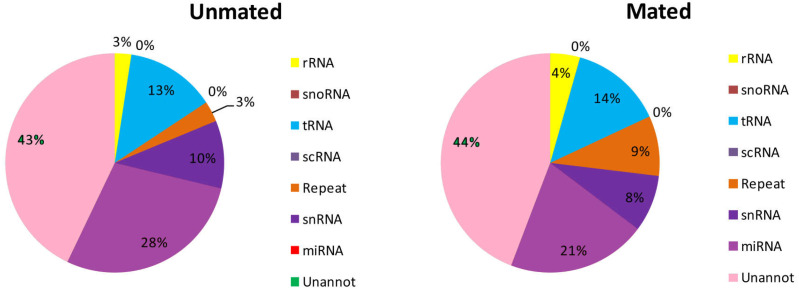
Distribution of small RNAs among different categories in unmated and mated libraries.

A total of 5,587,719 (28.75%) and 5,932,267 (35.27%) total reads in the unmated and mated libraries, respectively, were annotated as tRNA, rRNA, snRNA, snoRNA, scRNA, and repeats (Fig. 2, Table S2). Subsequently, the unannotated reads were mapped to the mature metazoan miRNAs in miRBase21.0 to identify known miRNAs in Chinese forest musk deer. A total of 5,512,277 and 3,455,159 reads were mapped to the mature metazoan miRNAs, which accounted for 28.36% and 20.54% of total clean reads in unmated and mated libraries, respectively. We identified 1,459 (distributed in 600 miRNA families) and 1,476 (distributed in 605 miRNA families) known miRNAs in the unmated and mated libraries, respectively (Table S3). Among the known miRNAs, 1,283 conserved miRNAs were present in both libraries and 369 miRNAs were detected in only one sample. Furthermore, we found that the count of reads for known miRNAs has large difference in the expression frequency between two libraries. In the unmated library, eleven miRNAs (bta-miR-26a, gga-miR-21-5p, chi-miR-21-5p, bta-miR-125b, ipu-miR-99b, bta-miR-27b, chi-miR-199b-3p, xtr-miR-26, bta-let-7g, bta-let-7b and chi-miR-29a-3p) were the dominant expressed miRNAs, with more than 100,000 reads (Table S3). However, In the mated library, bta-miR-145, gga-miR-21-5p, oar-miR-143, bta-miR-26a, chi-miR-21-5p and bta-miR-125b were predominately expressed miRNAs, each with more than 100,000 reads (Table S3).

Using the criteria for novel miRNA identification, we identified 45 novel miRNAs in the two libraries (Table S4) that we named “mbe-miR-new-N” (N =1–45). The novel mature miRNAs ranged in length from 20 to 24 nt, while the reads count was 6–937,639 in the two libraries.

### Differential expression analysis of known miRNAs

**Figure 3:**
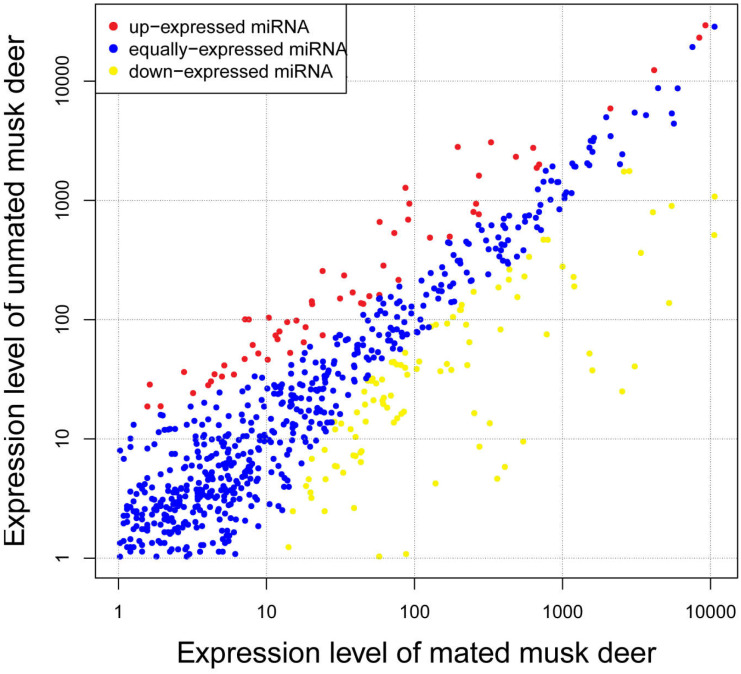
Differential expression profile of known miRNAs in unmated and mated musk deer.

To investigate differences in expression levels of known miRNA between unmated and mated individuals, we identified 174 known miRNAs that were differentially expressed between the two groups. Compared with miRNA expression in mated musk deer, 113 miRNAs were significantly down-regulated and 61 were significantly up-regulated in unmated musk deer (Fig. 3 and Table S5). Among the differentially expressed miRNAs, the fold-change of most miRNAs ranged from 1.5 to 4, with mdo-miR-206 having the highest fold-change (−7.49) in the unmated library compared with the mated library (Table S5). Notably, although chi-miR-21-5p, ipu-miR-99b and bta-miR-26a are up-regulated in unmated male, they are highly expressed in both libraries (Table 1).

**Table 1:** List of the 10 most differentially expressed miRNAs in unmated and mated males.

**microRNAs names**	**TPM (UM)**	**TPM (MM)**	**log2(FC)**	**Type**
bta-miR-26a	29,296.45	9,121.11	1.22	Most differentially expressed in both groups
chi-miR-21-5p	23,064.85	8,289.75	1.01	
ipu-miR-99b	12,327.71	4,106.88	1.12	
xtr-miR-26	5,879.14	2,080.29	1.03	Most differentially expressed in UM
ggo-miR-200b	3,059.92	324.53	2.77	
bta-miR-200c	2,799.67	193.54	3.39	
pma-miR-205a-5p	2,748.37	625.92	1.67	
bta-miR-21-5p	2,311.64	478.29	1.81	
bta-miR-195	1,993.76	685.68	1.07	
oar-miR-27a	1,868.73	660.89	1.03	
bta-miR-145	1,075.01	10,515.24	(3.76)	Most differentially expressed in MM
oar-miR-143	510.42	10,461.13	(4.82)	
ccr-miR-143	896.88	5,387.74	(3.05)	
bta-miR-1	137.38	5,183.03	(5.70)	
xtr-miR-22-3p	793.15	4,025.78	(2.81)	
dre-miR-145-5p	361.56	3,340.22	(3.67)	
xtr-miR-451	40.39	3,036.68	(6.70)	

### Target gene prediction and functional enrichment analysis

**Figure 4:**
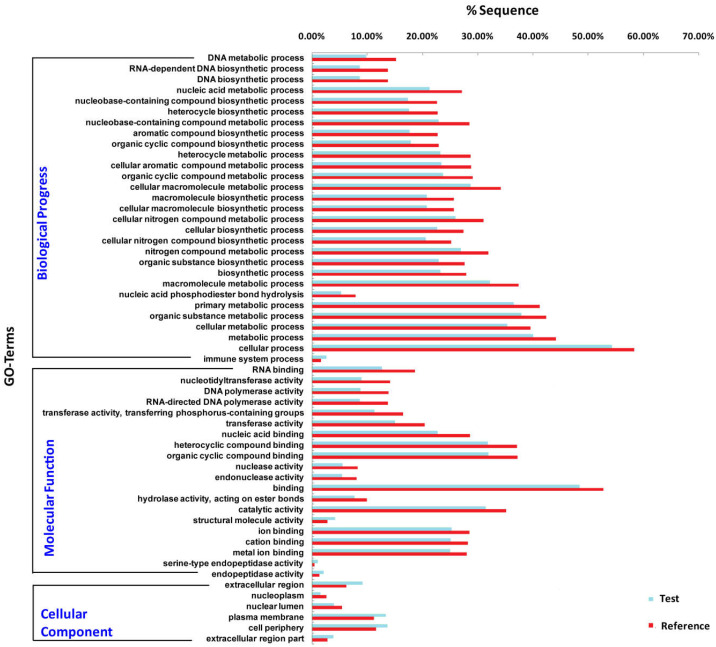
Functional enrichment for the putative target genes of the 10 most differentially expressed miRNAs.

To understand the role of miRNAs, it is necessary to predict the target genes of differentially expressed miRNAs. We obtained a total of 5,016 putative target genes for 174 differentially expressed miRNAs using miRanda (version 3.3a) and RNAhybrid (version 2.1.2) software. We further predicted the putative target genes of the 10 most expressed differentially expressed miRNAs, 782 and 714 target genes of UM and MM are obtained, respectively. Functional enrichment analysis showed that structure molecule activity, regulated exocytosis, regulation of secretion by cell, circulatory system process and regulation of proteolysis are more enriched in target genes of miRNA in unmated male. Some biological functions such as monosaccharide biosynthetic process, protein targeting, cellular protein catabolic process are more active in mated male (Fig. 4).

## Discussion

Although several studies have investigated the regulation of mammalian reproductive systems by miRNAs, these have mainly concentrated on model animal species and agricultural animals (Bjork et al., 2010; Tong et al., 2012; Yao et al., 2014; Lannes et al., 2015). To our knowledge, no research has yet been conducted on the role of miRNAs in musk deer. In the present study, we observed that most musk deer small RNAs were 20–24 nt in size, with 22 nt-length sequences dominating, which is consistent with the typical length of Dicer-processed small RNA products (Zhang, Stellwag & Pan, 2009). Interestingly, we also identified a small peak at 31–32 nt which has not been reported in other ruminant animals (Hossain et al., 2009; Ji et al., 2012). However, a similar phenomenon was seen in the large yellow croaker (*Pseudosciaena crocea*) (Qi et al., 2014), in which Qi et al. detected a primary peak at 20–24 nt and a secondary peak at 27–30 nt; this latter peak mainly derived from nuclear tRNA and rRNA. Therefore, it is possible that the 31–32 nt miRNAs in musk deer may also derive from nuclear tRNA and rRNA.

We identified 174 miRNAs are significantly differentially expressed between the unmated and mated males, among which chi-miR-21-5p, ipu-miR-99b and bta-miR-26a are highly expressed in both unmated and mated males. A total of 5,016 putative target genes are obtained, 782 and 714 target genes of UM and MM are predicted, respectively.

We also showed that chi-miR-21-5p was abundant in unmated (448,269 reads) and mated (139,420 reads) musk deer, and that it exhibited significant differential expression between the two groups. Carletti et al. reported that knockdown of miR-21 in ovaries increased granulosa cell apoptosis and decreased the ovulation rate (Carletti, Fiedler & Christenson, 2010). Another study showed that hsa-miR-21 expression was regulated by ovarian steroids in mouse granulosa cells and human endometrial stromal cells (Pan et al., 2007). In this study, *CHD7* was predicted as the target of miR-21-5p, mutations of this gene cause gonadal endocrine abnormalities, which manifests biochemically as hypogonadotropic hypogonadism (low sex steroid hormone and gonadotropin levels) (Kim et al., 2008; Neocleous et al., 2020; Xu et al., 2018). Furthermore, *CHD7* was enriched in “circulatory system process” and “regulation of secretion by cell”, suggesting that miR-21-5p might be crucial to endocrine function of musk gland in male musk deer.

MiR-143 was previously found to be highly expressed in mammalian gonads, indicating that it plays an important role in the mammalian reproductive system (Hossain et al., 2009; Xu et al., 2018; Landgraf et al., 2007; Mishima et al., 2008; Li et al., 2011). Its expression was reported to be under the negative control of follicle-stimulating hormone, which regulates folliculogenesis, and to gradually increase during follicular development (Yao et al., 2009). We found that miR-143 (including hsa-miR-143-5p, oar-miR-143, bta-miR-143, chi-miR-143-5p, lla-miR-143, and ccr-miR-143) was significantly down-regulated in unmated musk glands compared with mated musk glands. *HSD17B7*, as a target of miR-143, with higher expression level in testis than ovary, encodes sex-steroidogenic enzymes that is essential for testosterone metabolism (Nyuji et al., 2020; Kimoto et al., 2010). The participation of *HSD17B7* in regulation of secretion by cell testified the putative biological function of miR-143 in testosterone metabolism and musk secretion.

We also showed that the miR-200 family, including bta-miR-141, bta-miR-200a, and bta-miR-200c was significantly up-regulated in unmated musk glands compared with mated musk glands. Previous work suggested that the expression of miR-141, miR-200a, and miR-200c gradually decreased during primordial germ cell development (Hayashi et al., 2008), while another study reported significant increases in miR-141 within the seminal plasma of non-obstructive azoospermia patients compared with fertile controls (Wu et al., 2013). We predicted *Noc2* as a putative target of miR-141/200a, the encoded dual effector Noc2 has a crucial role in secretory-granule exocytosis both in endocrine and exocrine cells, secretory vesicles exocytosis exerts a variety of biological effects, it involves not only in the control of hormone exocytosis, but also in exocrine secretion including the determination of the size and shape of secretory granules (Cheviet, Waselle & Regazzi, 2004; Masanari et al., 2004; Kohichi et al., 2017). Musk gland’s seasonal development and musk secretion are regulated by sex hormones produced by the testis under the control of the hypothalamus-pituitary-testis system (Zhang et al., 2017), suggesting a possible involvement of Noc2.

Among the differentially expressed miRNAs, the muscle-specific miRNAs of pma-miR-133b-3p and cgr-miR-1 were identified in musk gland. This phenomenon was consistent with the findings of Huang’s study, they also identified miR-133 and miR-1 in the testis and ovary of Holstein Cattle (Huang et al., 2011). Intriguingly, these miRNAs have higher expression in mated library compared with unmated library. This result suggested that the previous study, which reported muscle -specific miRNAs, might be have some limits and need to be further verified (Mishima et al., 2008).

Functional enrichment provides an understanding of the roles of differentially expressed miRNA target genes. Our results illustrated that more targets were involved in “structure molecule activity”, “regulated exocytosis”, “regulation of secretion by cell”, “circulatory system process” and “regulation of proteolysis” for unmated male. Genes related to “monosaccharide biosynthetic process”, “protein targeting”, “cellular protein catabolic process” were more active in mated male. Which indicated that the most differentially expressed miRNAs were responsible for musk formation and secretion. Especially the pathway “regulation of secretion by cell” contains hormone secretion, hormone transport, establishment of protein localization to extracellular region, signal release etc. We deduced that miRNAs might be encapsulated by exosomes and secreted by musk gland cells, transported to target organs like testis through circulatory system.

The limited sample (*n* = 1) of each group was a vital shortcoming in our study due to the difficulty of sampling. Actually it was forbidden to sacrifice forest musk deer for sample collection. It is a rare protected animal and listed as an endangered species on the IUCN Red List. Due to the hardship of sample collection technical replicates and further validation experiments are indispensable to confirm our results.

## Conclusions

In summary, this study is the first to systematically characterize miRNA patterns of the musk gland from Chinese forest musk deer. We identified 1,652 known and 45 novel miRNAs from two libraries of UM and MM. Findings of functional enrichment suggest that miRNAs might affect musk yield in the male musk deer by regulating secretion and transport of hormones, which explains why unmated musk deer secrete more musk than mated musk deer. The putative miRNA-mRNA pairs (miR-21: *CHD7*, miR143: *HSD17B7*, miR-141/200a: *Noc2*) might involve in musk gland development and musk secretion, which need to be verified in our future study. These data will provide a comprehensive reference for further research into the roles of miRNAs in musk glands of musk deer, and will contribute to an understanding of the molecular mechanism of musk secretion.

## Supplemental Information

10.7717/peerj.12710/supp-1 Supplemental Information 1 Overview of small RNA deep sequencing data in musk glands of unmated and mated musk deer

10.7717/peerj.12710/supp-2 Supplemental Information 2 Total small RNA annotation

10.7717/peerj.12710/supp-3 Supplemental Information 3 Total known microRNAs in unmated and mated libraries

10.7717/peerj.12710/supp-4 Supplemental Information 4 Novel miRNAs predicted by miRDeep2

10.7717/peerj.12710/supp-5 Supplemental Information 5 MiRNAs showing differential expression between the two libraries

## Additional Information and Declarations

### Data Availability

The following information was supplied regarding data availability: The raw data of Illumina sequencing are available at NCBI SRA: SRR5378549 and SRR5366118.
